# A novel prognostic model based on six methylation-driven genes predicts overall survival for patients with clear cell renal cell carcinoma

**DOI:** 10.3389/fgene.2022.996291

**Published:** 2022-10-18

**Authors:** Hongmin Zhou, Tiancheng Xie, Yuchen Gao, Xiangcheng Zhan, Yunze Dong, Ding Liu, Yunfei Xu

**Affiliations:** ^1^ Department of Urology, Shanghai 10th People’s Hospital, Tongji University School of Medicine, Shanghai, China; ^2^ Department of Urology, Shanghai 10th People’s Hospital, Nanjing Medical University, Shanghai, China

**Keywords:** DNA methylation, clear cell renal cell carcinoma, nomogram, TCGA, prognostic model

## Abstract

Clear cell renal cell carcinoma (ccRCC) is a lethal urological malignancy. DNA methylation is involved in the regulation of ccRCC occurrence and progression. This study aimed to establish a prognostic model based on DNA methylation to predict the overall survival (OS) of patients with ccRCC. To create this model, we used the transcriptome and DNA methylation data of patients with ccRCC from The Cancer Genome Atlas (TCGA) database. We then used the MethylMix R package to identify methylation-driven genes, and LASSO regression and multivariate Cox regression analyses established the prognostic risk model, from which we derived risk scores. We incorporated these risk scores and clinical parameters to develop a prognostic nomogram to predict 3-, 5-, and 7-year overall survival, and its predictive power was validated using the ArrayExpress cohort. These analyses identified six methylation-driven genes (*SAA1*, *FUT6*, *SPATA18*, *SHROOM3*, *AJAP1*, and *NPEPL1*) that produced risk scores, which were sorted into high- and low-risk patient groups. These two groups differed in nomogram-predicted prognosis, the extent of immune cell infiltration, tumor mutational burden, and expected response to additional therapies. In conclusion, we established a nomogram based on six DNA methylation-driven genes with excellent accuracy for prognostic prediction in ccRCC patients. This nomogram model might provide novel insights into the epigenetic mechanism and individualized treatment of ccRCC.

## 1 Introduction

Renal cell carcinoma is a common malignant tumor originating from the renal parenchymal urothelial system, and more than 70% of renal cell carcinomas are renal clear cell carcinomas (Jonasch et al., 2021). Surgery is the main treatment for localized ccRCC, but 20%–40% of patients experience postoperative recurrence or metastasis (Teng et al., 2014). The 5-year survival rate of patients with ccRCC and distant metastases is extremely low, and the prognosis is poor (Atkins and Tannir, 2018). Treatment of advanced and metastatic ccRCC relies primarily on immunotherapy, targeted therapy, and chemotherapy (Atkins and Tannir, 2018), but not all patients can benefit from these treatments. Therefore, early identification of patients with high-risk and improved treatment decisions are expected to increase overall survival in patients with ccRCC. Presently, the TNM staging system cannot well characterize the biological heterogeneity of tumors, So, its performance in predicting the prognosis of ccRCC needs to be improved (Ljungberg et al., 2015). Exploring biomarkers with prognostic value for ccRCC patients is critical for optimizing treatment decisions. Epigenetic changes are highly involved in cancer progression (Jones and Baylin, 2007). DNA methylation is an important aspect of epigenetic status, and it is involved in transcriptional regulation and maintenance of genomic stability (Pu et al., 2016). Identifying abnormal changes in DNA methylation can be used for cancer risk evaluation, early diagnosis, and prognostic prediction (Liu et al., 2021). Hypermethylation of promoter or enhancer CpG regions in ccRCC can lead to inactivation of important tumor suppressor genes, such as *SFRP1* (Morris et al., 2010), *RASSF1A* (Morrissey et al., 2001), and *STK11* (Zheng et al., 2017). Aberrant DNA methylation may influence the occurrence and progression of ccRCC. Several studies have reported the prognostic significance of DNA aberrant methylation in ccRCC (Deckers et al., 2015; Zhao et al., 2016; Fabrizio et al., 2017). However, there has yet to be a comprehensive and systematic methylation assessment in patients with ccRCC to further explore the role of dysregulated DNA methylation and corresponding gene expression changes, which can inform prognosis and treatment decisions in patients with ccRCC.

MethylMix is an R-based algorithm for identifying differentially methylated genes in specific diseases (Cedoz et al., 2018). In this study, DNA methylation and gene expression data from patients with ccRCC were obtained from The Cancer Genome Atlas (TCGA) database, and methylation-driven genes were identified using the MethylMix R package. A six DNA methylation risk scoring model was constructed using LASSO regression analysis. We combined the risk score with clinicopathological risk factors to construct a nomogram for ccRCC patients’ overall survival prediction and validated the model using the ArrayExpress database. In addition, we explored the molecular and immune characteristics and drug sensitivity of the risk assessment groups to provide new insights into their roles in ccRCC and new avenues of therapeutic research. Our findings suggest that the six gene risk model can achieve accurate prediction of prognosis in ccRCC.

## 2 Materials and methods

### 2.1 Data acquisition and processing

In this study, RNA-sequencing data (including 71 non-cancerous tissues and 512 ccRCC tissues), gene mutations, and clinicopathological data for ccRCC were downloaded from the TCGA database. TCGA-Assembler2 was used to acquire level-3 methylation data (including 24 non-cancerous tissues and 302 ccRCC tissues) using the Illumina Infinium Human Methylation 450 platform (Wei et al., 2018). We used the E-MTAB-1980 dataset (N = 101) from the ArrayExpress database as the external validation cohort.

### 2.2 Identification of methylation-driven genes

The MethylMix R package was used to analyze DNA methylation data and paired gene expression data of patients with ccRCC to obtain MDGs. The MethylMix analysis requires three steps. First, correlation analysis was performed on gene methylation and paired gene expression data to obtain transcriptionally predicted genes. Next, a β-mixture model was established for each gene. Finally, the methylation status was compared between 302 ccRCC samples and 24 non-cancerous samples using the Wilcoxon rank test to obtain differentially methylated genes.

### 2.3 Functional enrichment and pathway analysis of MDGs

To understand the potential molecular mechanisms of MDGs, Gene Ontology (GO) enrichment analysis was performed using the clusterProfiler R package.

### 2.4 Building and validating of the risk score model

Independent MDGs that were significantly related to prognosis were identified using least absolute shrinkage and selection operator (LASSO) regression analysis. A risk score prediction model with six genes was obtained by weighting mRNA expression levels using multivariate Cox regression coefficients. We divided the patients with ccRCC into two groups according to the median risk score: high- and low-risk. We conducted Kaplan–Meier curve and time-dependent receiver operating characteristic curve (tdROC) analyses to measure the model’s predictive performance.

### 2.5 Establishment and validation of the predictive nomogram

Univariate and multivariate Cox regression analyses were performed to assess the significance of the risk scoring model and other traditional clinical features for predicting overall survival (OS) for patients with ccRCC. A prognostic nomogram for patients with ccRCC was constructed based on risk scores and other traditional clinical parameters to predict the 3-, 5-, and 7-year OS. The consistency index (C-index) was calculated to quantify the discriminative performance of the nomograms. The nomogram generated a prognostic risk score for each patient. The predictive performance of the nomogram was further assessed using the tdROC curve analysis. A calibration plot was constructed to compare the clinically observed and predicted survival rates. We validated the nomogram using a dataset obtained from ArrayExpress.

#### 2.6 Weighted gene correlation network analysis and gene set enrichment analysis

Weighted gene correlation network analysis (WGCNA) was performed to identify significant modules related to the six gene risk score using the R package WGCNA. The construction process was the same as previously described (Langfelder and Horvath, 2008). Module Membership (MM) was defined as the Pearson’s correlation between gene expression and module eigengenes. Gene significance (GS) was defined as the Pearson’s correlation between gene expression and certain clinical trait. Based on cutoff criteria |MM|>0.8, |GS|>0.7, hub genes were identified in the significant modules. Differentially expressed genes (DEGs) between the two groups with high- and low six-gene score were identified using Deseq2 (Anders and Huber, 2010). The biological processes in the two risk subgroups were explored using gene set enrichment analysis (GSEA) based on the GO Biological Processes with the clusterProfiler package of R (Yu et al., 2012).

### 2.7 Gene mutation analysis and antitumor drug sensitivity analysis

Gene mutation information was downloaded from the TCGA database. The mutations in each ccRCC sample were calculated using maftools R package (Mayakonda et al., 2018). GDSC (https://www.cancerrxgene.org/) is a public online database that provides information on molecular markers of drug sensitivity and response in cancer cells, providing a unique resource for facilitating the discovery of new targets for cancer therapy (Yang et al., 2013). We used GDSC to explore differences in antitumor drug sensitivity between the two risk subgroups and the oncopredict package of the R program was used for analysis. Lower scores in the analysis represent higher drug sensitivity.

### 2.8 Tumor-infiltrating immune cells characteristics and tumor immune dysfunction and exclusion score

CIBERSORT analysis was used to assess the relative proportion of 22 tumor-infiltrating immune cells in ccRCC tumor tissues. Wilcoxon rank-sum test was used for analyzing the different levels of immune cell infiltration between the two risk groups. The response of different risk groups to immunotherapy was predicted using the TIDE (http://tide.dfci.harvard.edu/) algorithm.

### 2.9 Cell culture

ccRCC cell lines (SW839, A498) and the human embryonic kidney cell line, 293-T, were obtained from the Cell Bank of the Chinese Academy of Sciences. SW839 cells and A498 cells were cultured in RPMI-1640 (Gibco) containing 10% fetal bovine serum (Gibco). The 293-T cell line was cultured in DMEM medium (Gibco) containing 10% fetal bovine serum (Gibco). All mediums were treated with 100 U/ml penicillin and 100 μg/ml streptomycinm, and all cells were incubated at 37°C with 5% CO2.

### 2.10 Quantitative real-time PCR (qRT-PCR)

Using the ESscience RNA-Quick Purification Kit (YiShan Biotech, China) to extract the total RNA following the manufacturer’s instructions. The cDNA was synthesized with the PrimeScript RT reagent Kit with gDNA Eraser (Takara, Japan). qRT-PCR was performed using the TB Green Premix Ex Taq II (Takara, Japan) in ABI Quantstudio Dx Real-Time PCR instrument. The primers were listed in Supplementary Table S1. Relative mRNA levels were normalized using GAPDH as an internal control. The 2^−ΔΔCt^ method was applied for analysis of the results.

### 2.11 Statistical analysis

The Wilcoxon rank-sum test was used to compare differences between the two groups. Survival curves of the groups were plotted using the Kaplan–Meier method and compared using the log-rank test. Variables associated with OS were analyzed using univariate Cox and multivariate Cox regressions analyses. All data analyses were performed using R version 4.0.3. A two-tailed *p*-value of less than 0.05 was considered significant.

## 3 Results

### 3.1 Identification of MDGs in ccRCC

**FIGURE 1:**
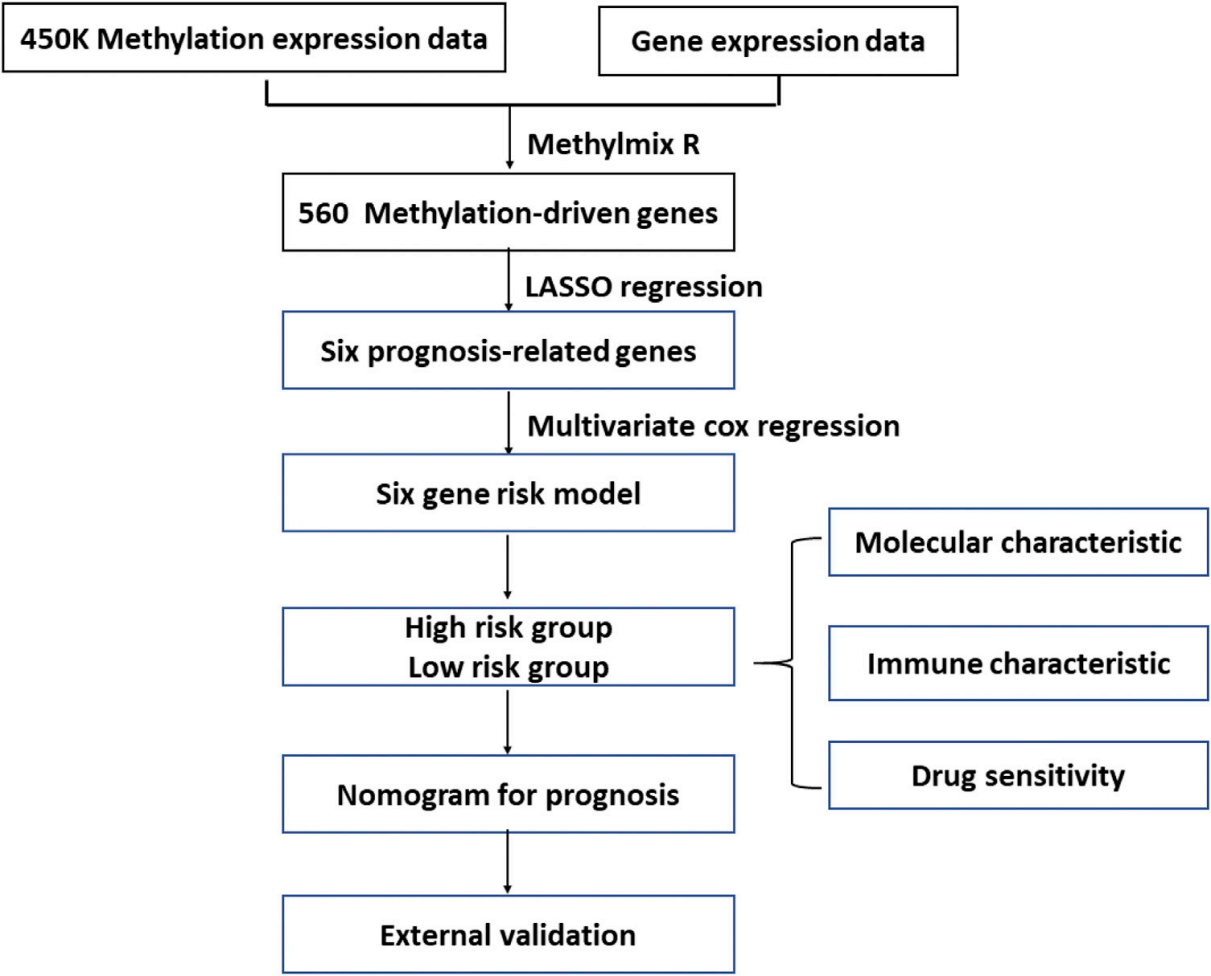
The flowchart of identification and analysis of methylation-driven genes in ccRCC.

**FIGURE 2:**
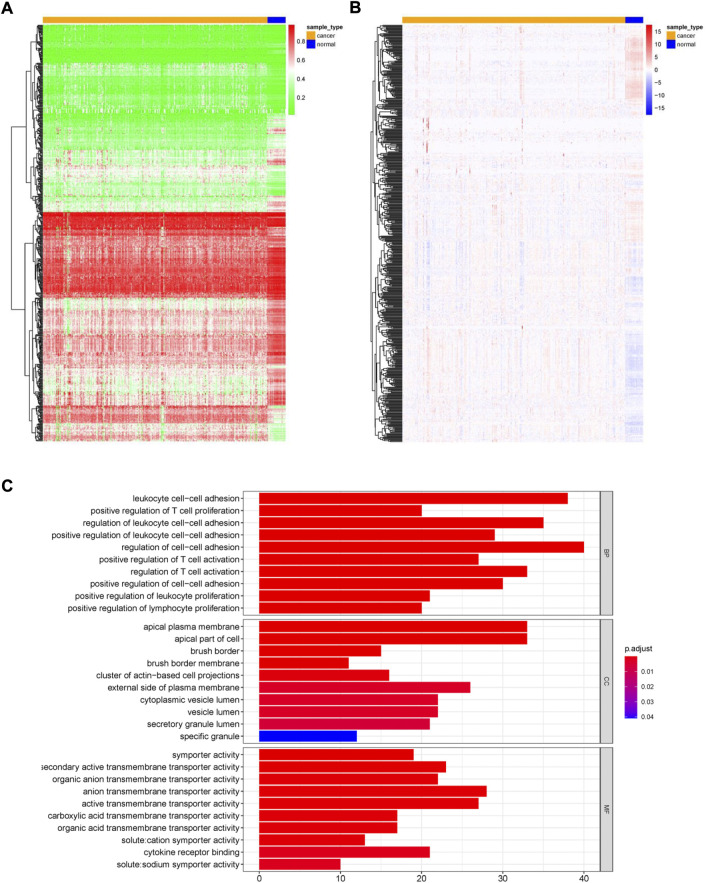
Overview of methylation driven genes in ccRCC. **(A)** Heatmap of methylation levels of 560 methylation-driven genes. **(B)** Heatmap of gene expression levels of 560 methylation-driven genes. **(C)** GO analysis of 560 methylation-driven genes.

This study’s workflow is presented in Figure 1. To identify MDGs in ccRCC, we performed MethylMix analysis on 302 ccRCC and 24 non-cancerous samples, resulting in a total of 560 MDGs. Heatmaps of the methylation and gene expression levels of these 560 MDGs were shown in Figures 2A,B. GO analysis showed that the enriched biological processes included regulation of cell-cell activation, positive regulation of lymphocyte proliferation, positive regulation of T cell proliferation, and leukocyte cell-cell adhesion. Markedly enriched cellular components included the apical plasma membrane, external components of the plasma membrane, and apical part of the cell. The top three terms of molecular function included “anion transmembrane transporter activity,” “active transmembrane transporter activity,” and “symporter activity” (Figure 2C).

### 3.2 Generation of a prognostic risk score model for ccRCC

**FIGURE 3:**
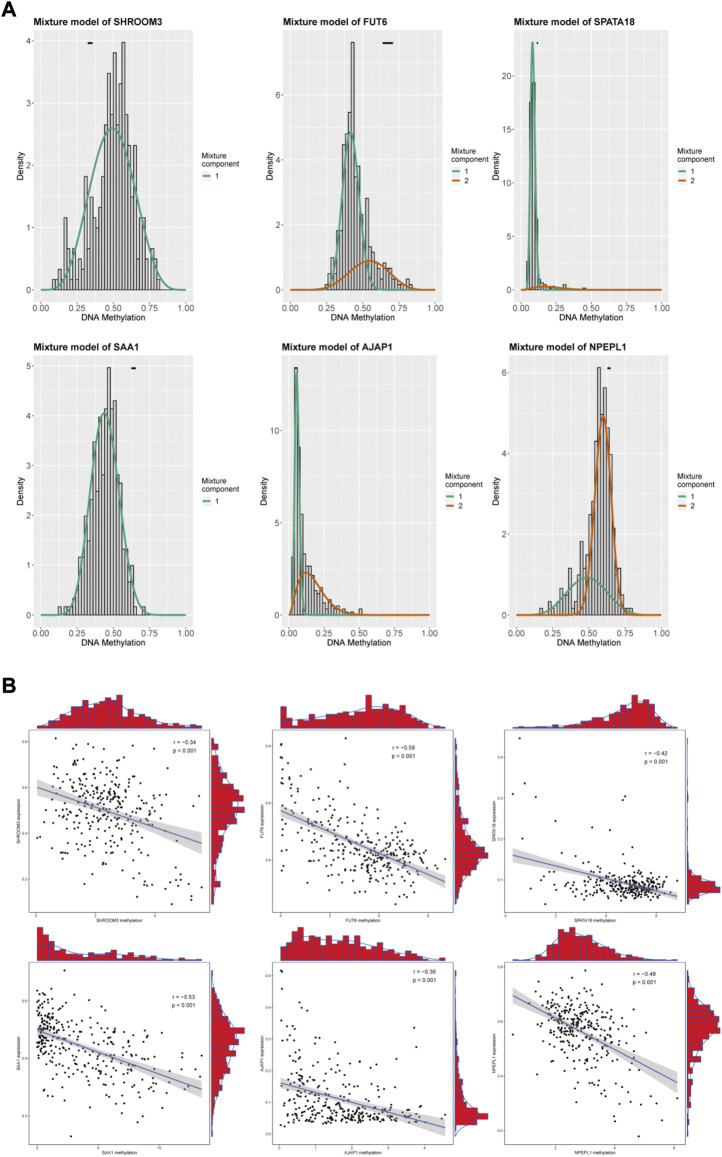
Summary of the Six methylation-driven genes. **(A)** The distribution map showing the methylation degree of methylation-driven genes in ccRCC. **(B)** Regression analysis between gene expression and DNA methylation.

**FIGURE 4:**
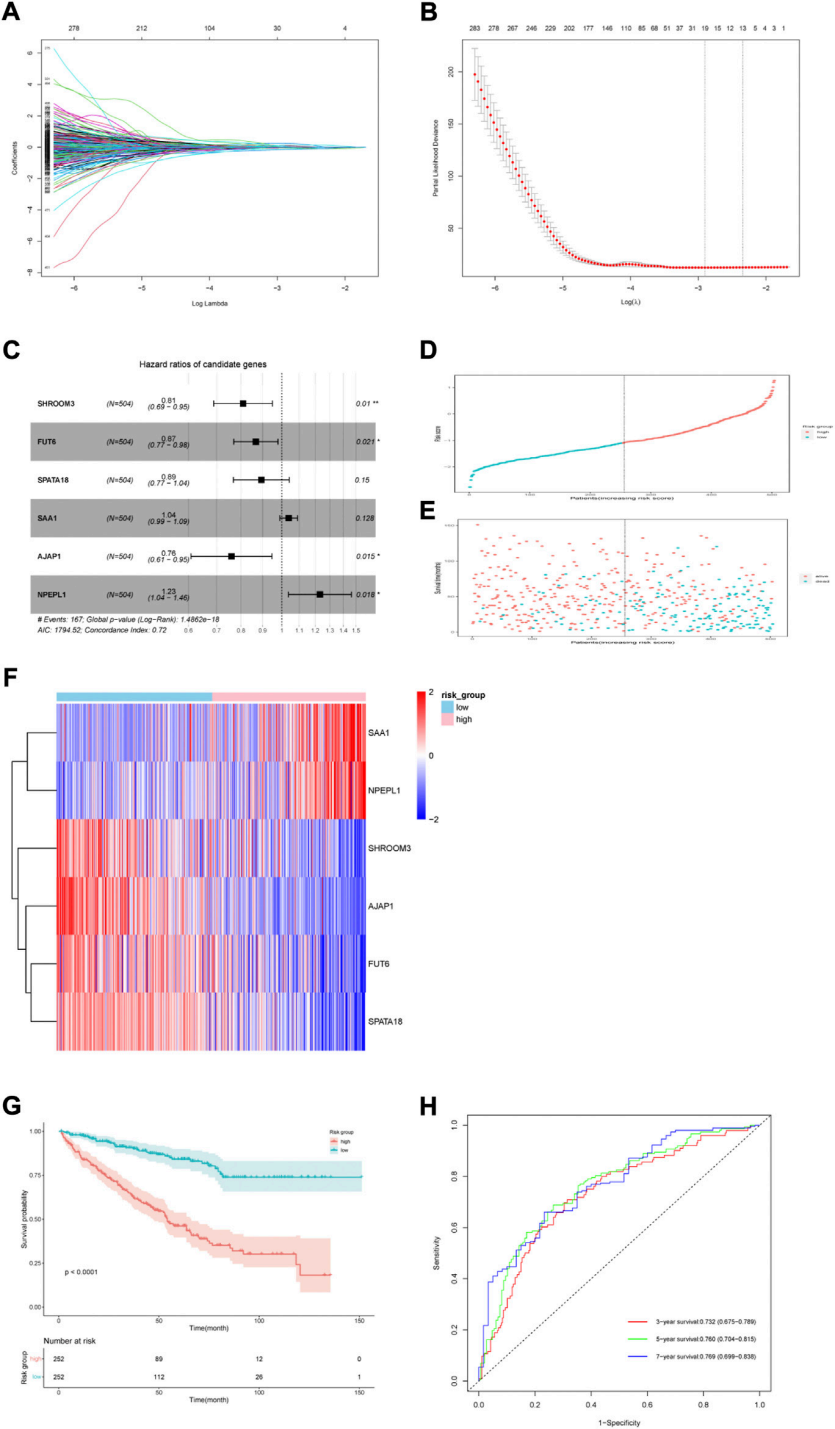
Six-gene risk score model construction in the TCGA cohort. **(A)** LASSO coefficients. **(B)** 10-time cross validation for tuning parameter selection by LASSO regression. **(C)** Multivariable Cox proportional hazard model of six genes. **(D)** Risk score distribution of high-risk and low-risk patients. **(E)** Survival status scatter plots for high-risk and low-risk patients. **(F)** Heatmap of nine genes in the two risk groups. **(G)** Kaplan–Meier (KM) estimate of the overall survival (OS) in the two risk groups. **(H)** The time-dependent ROC curves for 3-, 5- and 7-year survival prediction.

We included 560 genes in the LASSO regression analysis and considered genes that appeared more than 700 times out of 1,000 repeats as candidate genes. Six DNA methylation-driven genes (*SAA1*, *SHROOM3*, *FUT6*, *SPATA18*, *AJAP1*, and *NPEPL1*) were selected as prognostic genes. *SAA1* and *NPEPL1* were hypomethylated, whereas *SHROOM3*, *FUT6*, *SPATA18*, and *AJAP1* were hypermethylated (Figure 3A). The expression levels of the six genes were significantly negatively correlated with methylation (Figure 3B). A risk model was established using the regression coefficients from the multivariate Cox regression analysis (Figures 4A–C). The risk score for each patient was calculated as follows:

Risk score=(−0.212×SHROOM3)+(−0.142×FUT6)+(−0.112×SPATA18)+(0.038×SAA1)+(−0.275×AJAP1)+(0.209×NPEPL1).

For 504 patients with complete clinical data, we divided them into two groups based on the median six gene risk score: high-risk (*n* = 252) and low-risk (*n* = 252) (Figure 4D). The clinical features of the patients are presented in Table 1. The relationship between the risk score, survival status, and survival time is presented in Figure 4E. A heatmap of the expression levels of these six genes is shown in Figure 4F. Kaplan–Meier analysis of both groups showed a worse OS in the group with high risk (Figure 4G). The AUCs of the model at 3-, 5-, and 7-year were 0.732 (0.675–0.789), 0.760 (0.704–0.815), and 0.769 (0.699–0.838), respectively (Figure 4H).

**TABLE 1:** The clinicopathological characteristics of patients in the TCGA cohort and ArrayExpress cohort.

Variables	Training set (*n* = 504)	Validation set (*n* = 101)
Age [n (%)]		
Median	60	64
>65 years	171 (33.9%)	44 (43.6%)
≤65 years	333 (66.1%)	57 (56.4%)
Gender [n (%)]		
Male	334 (66.3%)	77 (76.2%)
Female	170 (33.7%)	24 (23.8%)
Grade [n (%)]		
1	12 (2.4%)	13 (12.9%)
2	220 (43.7%)	59 (58.4%)
3	200 (39.6%)	22 (21.8%)
4	72 (14.3%)	5 (4.9%)
undetermined		2 (2.0%)
T stage [n (%)]		
T1	258 (51.2%)	68 (67.3%)
T2	66 (13.1%)	11 (10.9%)
T3	169 (33.5%)	21 (20.8%)
T4	11 (2.2%)	1 (1.0%)
N stage [n (%)]		
N0	226 (44.8%)	94 (93.1%)
N1	14 (2.8%)	7 (6.9%)
NX	264 (52.4%)	
M stage [n (%)]		
M0	403 (79.9%)	89 (88.1%)
M1	77 (15.3%)	12 (11.9%)
MX	24 (4.8%)	
Mean overall survival (months)	46	59

### 3.3 Establishment and assessment of a nomogram for OS prediction in ccRCC

**FIGURE 5:**
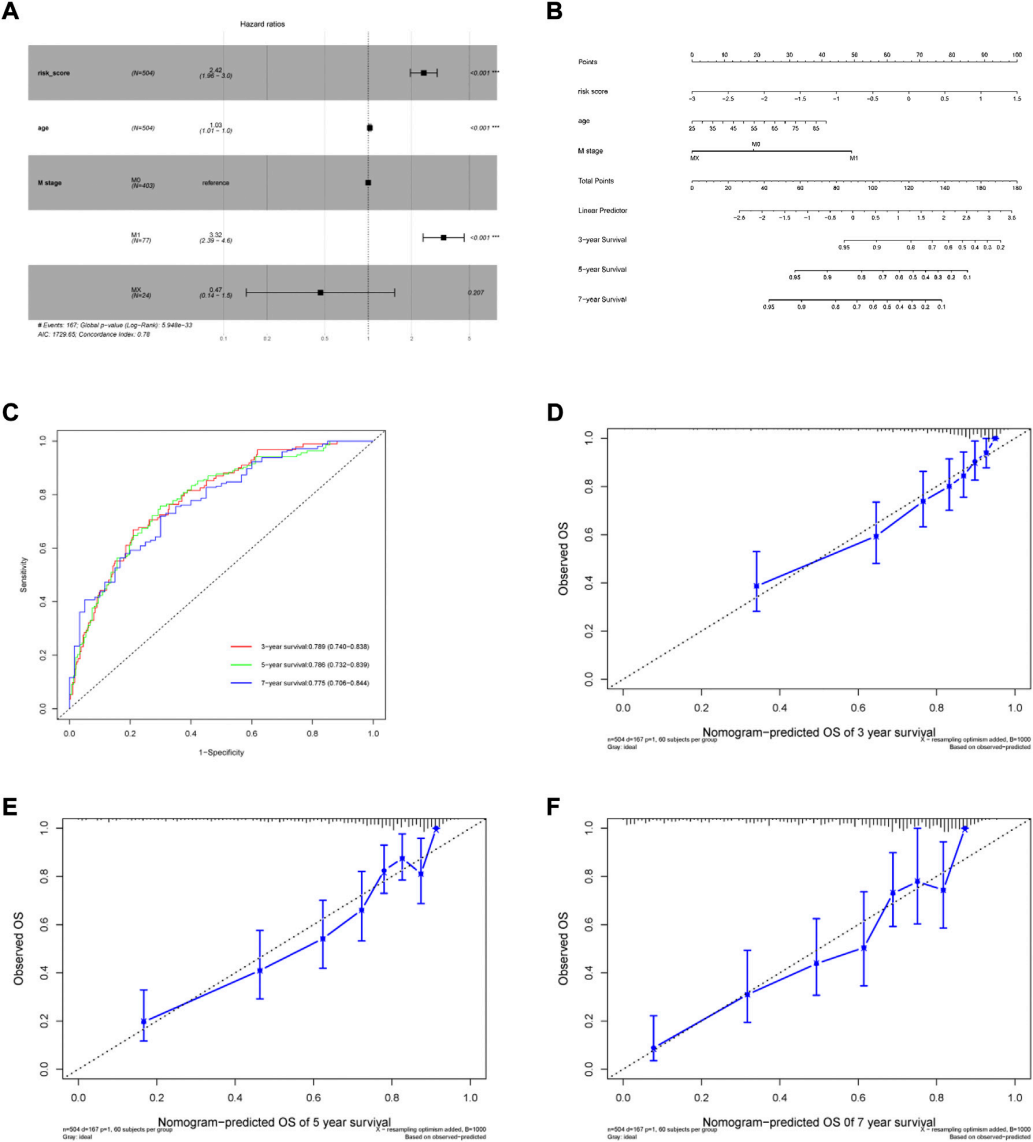
Generation of the nomogram incorporating risk score and clinical parameters. **(A)** Multivariable Cox proportional hazard model. **(B)** Nomogram for predicting 3-, 5- and 7-year overall survival of ccRCC patients. **(C)** The time-dependent ROC curves of the nomogram for 3-, 5- and 7-year survival prediction. **(D–F)** Calibration curves of the nomogram prediction of 3-, 5- and 7-year OS of ccRCC patients.

Patient age, M stage, and risk score were considered as significant predictors after univariate and multivariate Cox regression analyses (Table 2). We quantified these predictors to establish a prognostic nomogram (Figures 5A,B). The C-index of the nomogram is 0.776. According to tdROC analysis, the AUCs of the nomogram at 3-, 5-, and 7-year were 0.789 (0.740–0.838), 0.786 (0.732–0.839), 0.775 (0.706–0.844), respectively (Figure 5C). The calibration curve showed consistency between the nomogram OS prediction and actual survival rate (Figures 5D–F).

**TABLE 2:** Cox regression analyses in TCGA cohort.

		Univariable cox regression				Multivariable cox regression			
		95% CI				95% CI			
		HR	Lower	Upper	*P*	HR	Lower	Upper	*P*
Risk score	2.709	2.234	3.284	<0.001	2.124	1.679	2.686	<0.001	
Age		1.028	1.015	1.042	<0.001	1.026	1.012	1.041	<0.001
Gender	female	References							
	male	0.952	0.694	1.307	0.762				
T stage	1.912	1.619	2.258	<0.001	1.138	0.924	1.402	0.224	
N stage	N0	References				References			
	N1	3.287	1.699	6.355	<0.001	1.342	0.668	2.697	0.408
	NX	0.813	0.594	1.112	0.195	0.826	0.599	1.137	0.241
M stage	M0	References				References			
	M1	4.445	3.239	6.102	<0.001	2.818	1.926	4.123	<0.001
	MX	1.005	0.318	3.181	0.993	0.539	0.163	1.786	0.312
Grade	2.675	1.89	3.786	<0.001	1.279	0.861	1.901	0.223	

### 3.4 External validation of the nomogram

**FIGURE 6:**
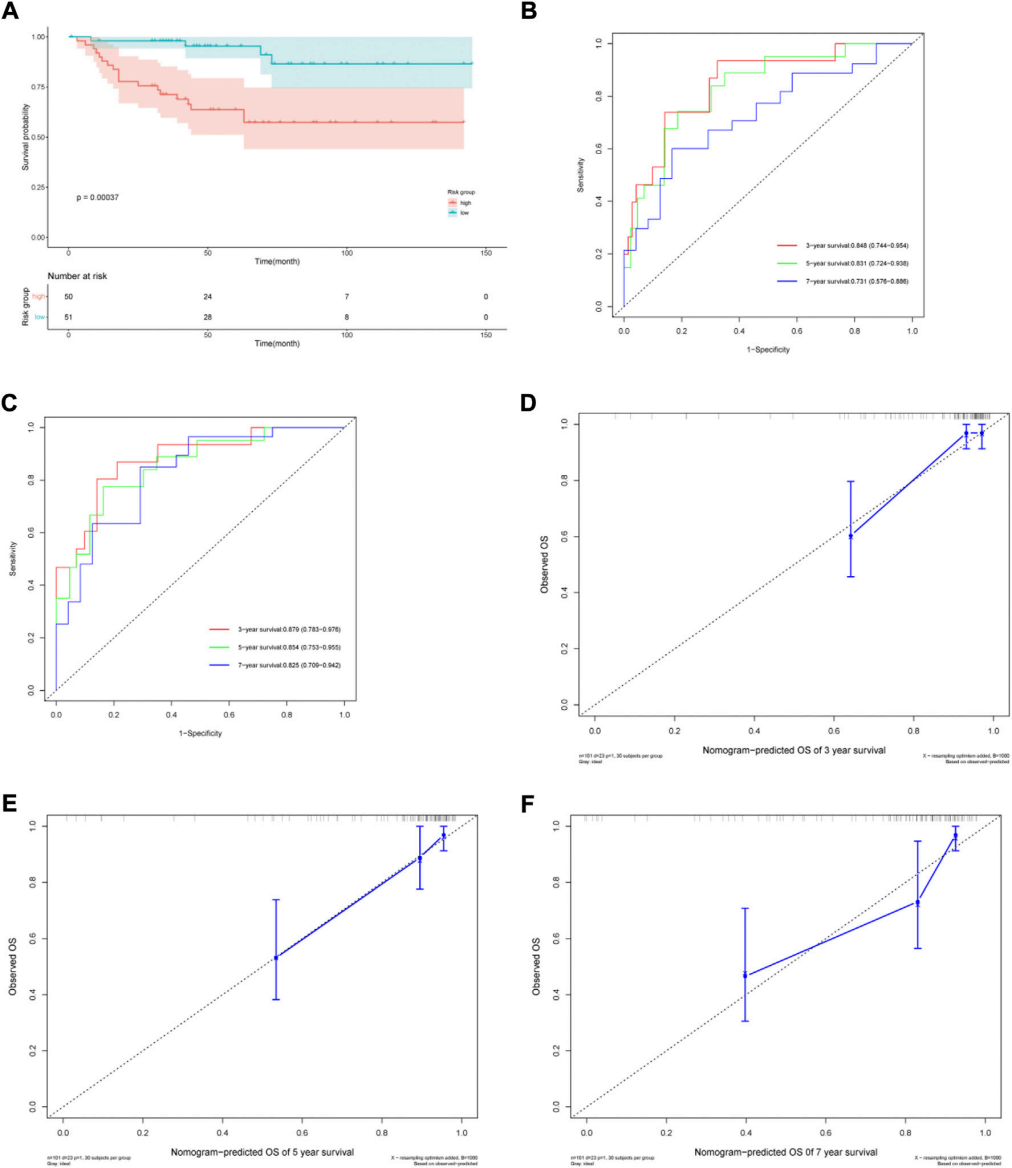
Validation of the prognostic risk model and nomogram. **(A)** Kaplan–Meier (KM) estimate of the overall survival (OS) in the two risk groups in the validation cohort. **(B)** The time-dependent ROC curves of the risk model for 3-, 5- and 7-year survival prediction in the validation cohort. **(C)** The time-dependent ROC curves of the nomogram for 3-, 5- and 7-year survival prediction in the validation cohort. **(D–F)** Calibration curves of the nomogram prediction of 3-, 5- and 7-year OS of ccRCC patients in the validation cohort.

The ArrayExpress dataset E-MTAB-1980 was used as an external dataset to validate the risk model and nomogram. Risk score was considered as a significant predictor after univariate and multivariate Cox regression analyses in the E-MTAB-1980 cohort. (Supplementary Table 2). Similar to the TCGA cohort, Kaplan–Meier analysis indicated poorer OS in the high-risk group (Figure 6A). The risk model AUCs for predicting the 3-, 5-, and 7-year OS in the validation set were 0.848 (0.744–0.954), 0.831 (0.724–0.938), 0.731 (0.576–0.886), respectively (Figure 6B). The nomogram AUCs for predicting 3-, 5-, and 7-year OS in the validation set were 0.879 (0.783–0.976), 0.854 (0.753–0.955), and 0.825 (0.709–0.942), respectively (Figure 6C). The calibration curves showed high consistency between the predicted and observed values (Figures 6D–F). Our results show that the predictive risk model and nomogram performed well in the validation set.

### 3.5 Molecular characteristics of different risk groups

**FIGURE 7:**
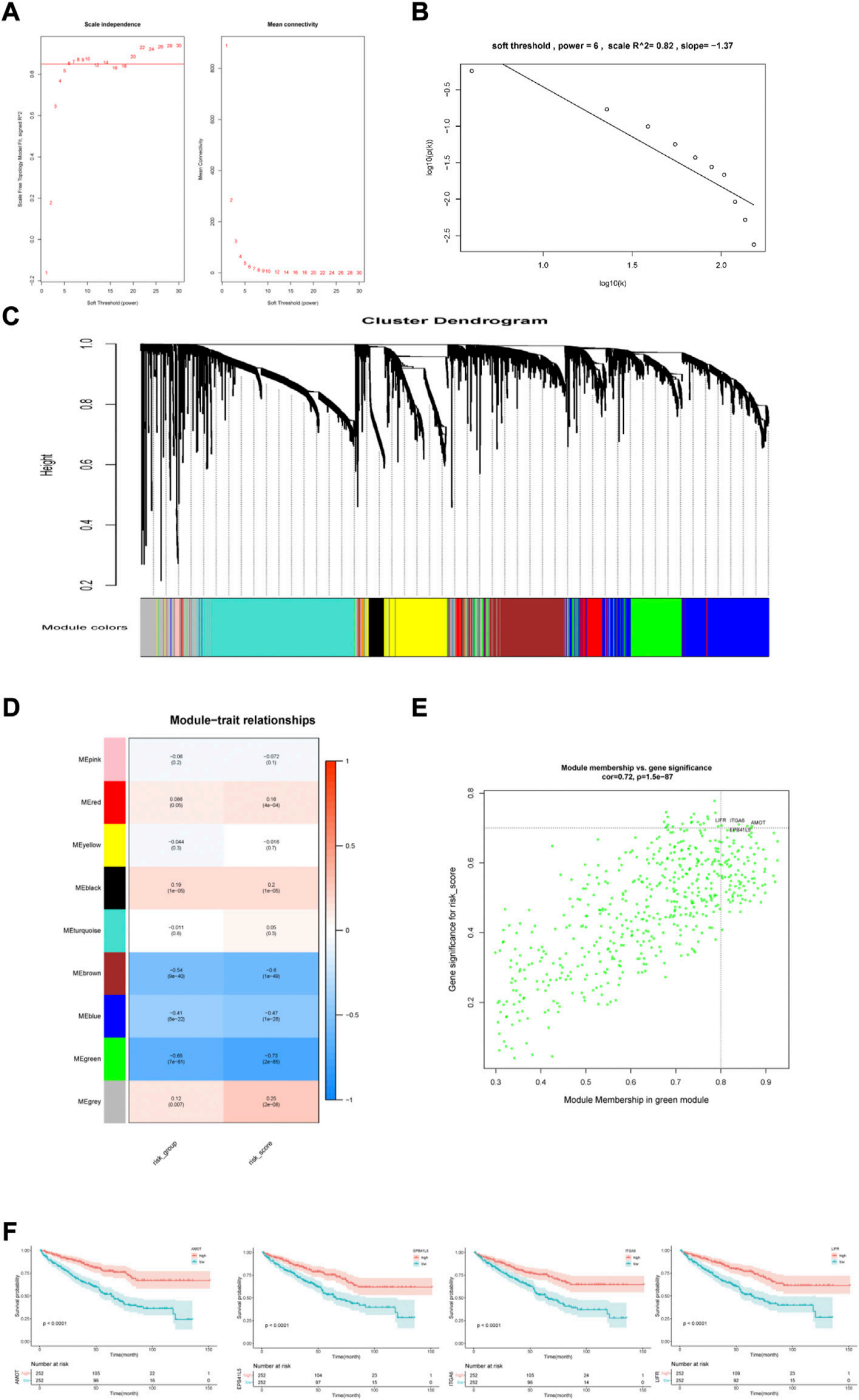
Construction and analysis of weighted gene correlation network. **(A,B)** Soft threshold filtering and validation. **(C)** The cluster dendrogram of genes. **(D)** The relationship between 9 module and the risk model. **(E)** Identification of the hub genes (|MM|>0.8, |GS|>0.7). **(F)** KM analysis for 4 hub genes.

To further investigate the different characteristics of the two risk subgroups, we performed WGCNA and GSEA analyses. We included genes with the top 5,000 median absolute deviations in the WGCNA analysis, and the soft threshold parameter was set to β = 6 (R2 = 0.82) (Figures 7A,B). Nine co-expressed gene modules were identified using average linkage hierarchical clustering (Figure 7C). We found that the green module was most relevant to the risk score (Cor = 0.65, p = 7e-61 for the risk group; Cor = 0.73, p = 2e-85 for the risk score) (Figure 7D). The green module contained 541 genes. Setting |MM| >0.8 and |GS| >0.7, the high connectivity of the four hub genes (*LIFR*, *ITGA6*, *AMOT*, and *EPB41L5*) in the significant module was determined (Figure 7E). KM analysis showed that higher expression levels of the four genes indicated a favorable prognosis (Figure 7F).

**FIGURE 8:**
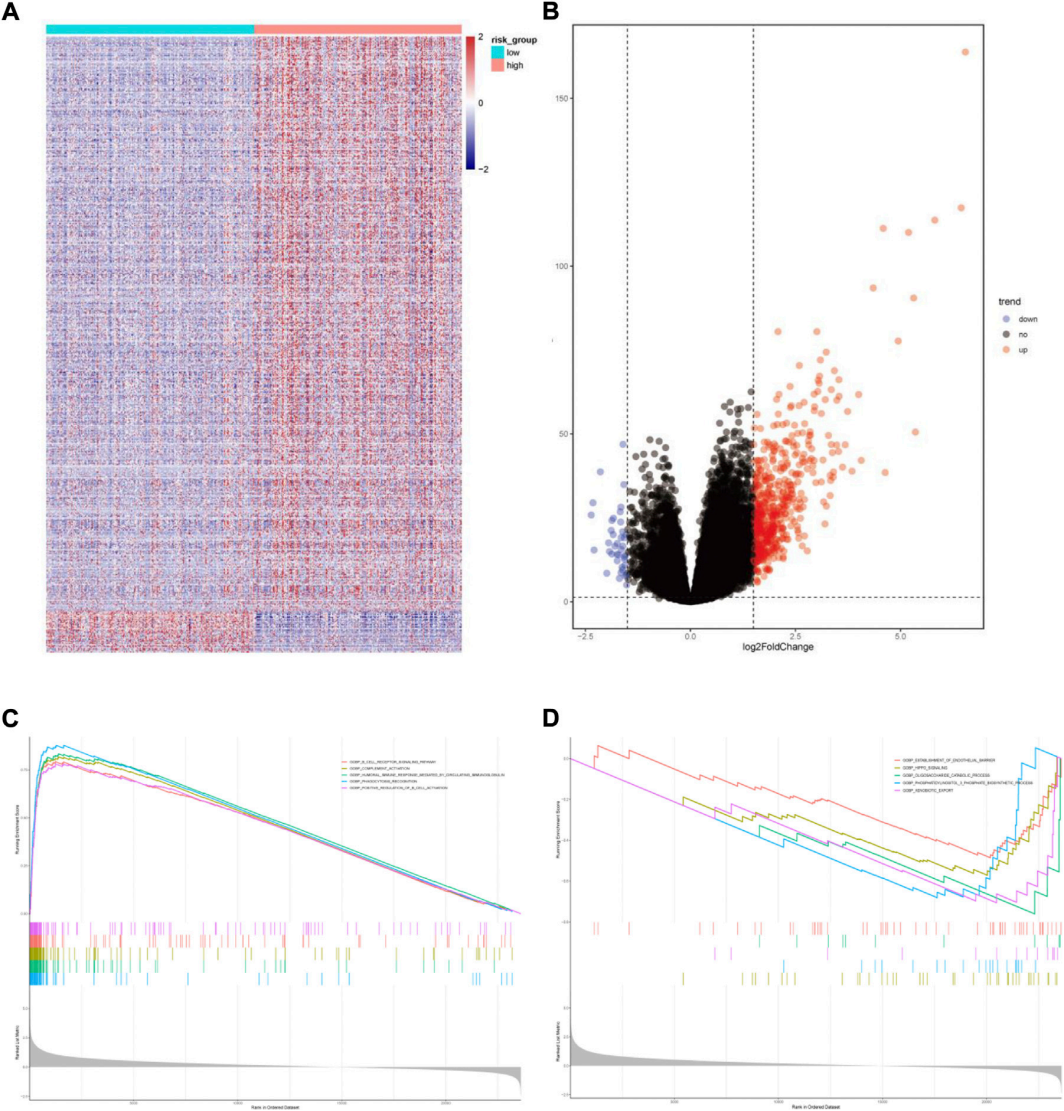
Functional enrichment analysis of differentially expressed genes between the two risk groups. **(A)**Heatmap of differentially expressed genes. **(B)** Volcano map of differentially expressed genes. **(C)** Gene sets enriched in high-risk group. **(D)** Gene sets enriched in low-risk group.

We also performed GSEA analysis in the two risk groups. Setting the absolute value of normalized enrichment score (NES) > 1.5 and the false discovery rate (FDR) < 0.05, we identified 591 DEGs between the two risk groups with 552 up-regulated and 39 down-regulated genes (Figures 8A,B). GO enrichment analysis showed that some biological processes, such as the B cell receptor signaling pathway, complement activation, humoral immune response mediated by circulating immunoglobulin, phagocytosis recognition and positive regulation of B cell activation were enriched in the high-risk group. The low-risk group was mainly associated with establishing the endothelial barrier, Hippo signaling, oligosaccharide catabolism, phosphatidylinositol-3-phosphate biosynthesis, and xenobiotic export processes (Figures 8C,D).

**FIGURE 9:**
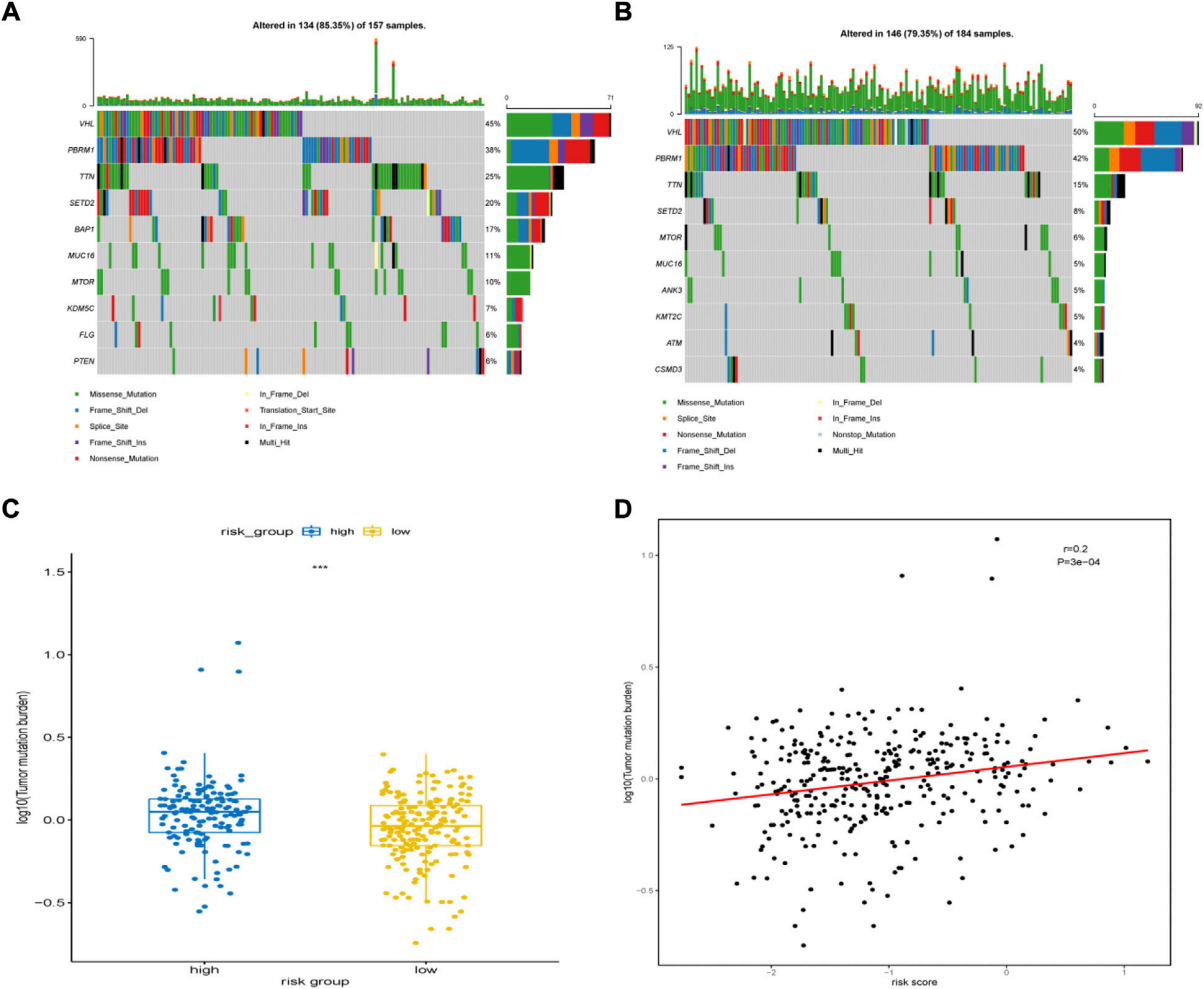
Somatic mutation features between high-risk group and low-risk group. **(A)** The top 10 most significantly mutated genes in the high-risk group. **(B)** The top 10 most significantly mutated genes in the low-risk group. **(C)** Difference in TMB between the high- and low-risk groups. **(D)** Correlation between TMB levels with risk-scores.

We then analyzed the landscape of somatic mutations in the two risk groups. We identified the top ten genes with the highest mutation rates in both groups (Figures 9A,B). The mutation rates of *VHL*, *PBRM1*, *TTN*, and *SETD2* in both the groups exceeded 20%. Common mutations in the high-risk group also included *BAP1*, *KDM5C*, *FLG*, and *PTEN*, whereas common mutations in the low-risk group included *ANK3*, *KMT2C*, *ATM*, and *CSDM3*. In addition, patients in the group with high risk had a higher tumor mutational burden (TMB) (Figure 9C). The risk score was positively correlated with TMB (r = 0.2, P = 3e-04, Figure 9D)

### 3.6 Immune landscape of different subgroups

**FIGURE 10:**
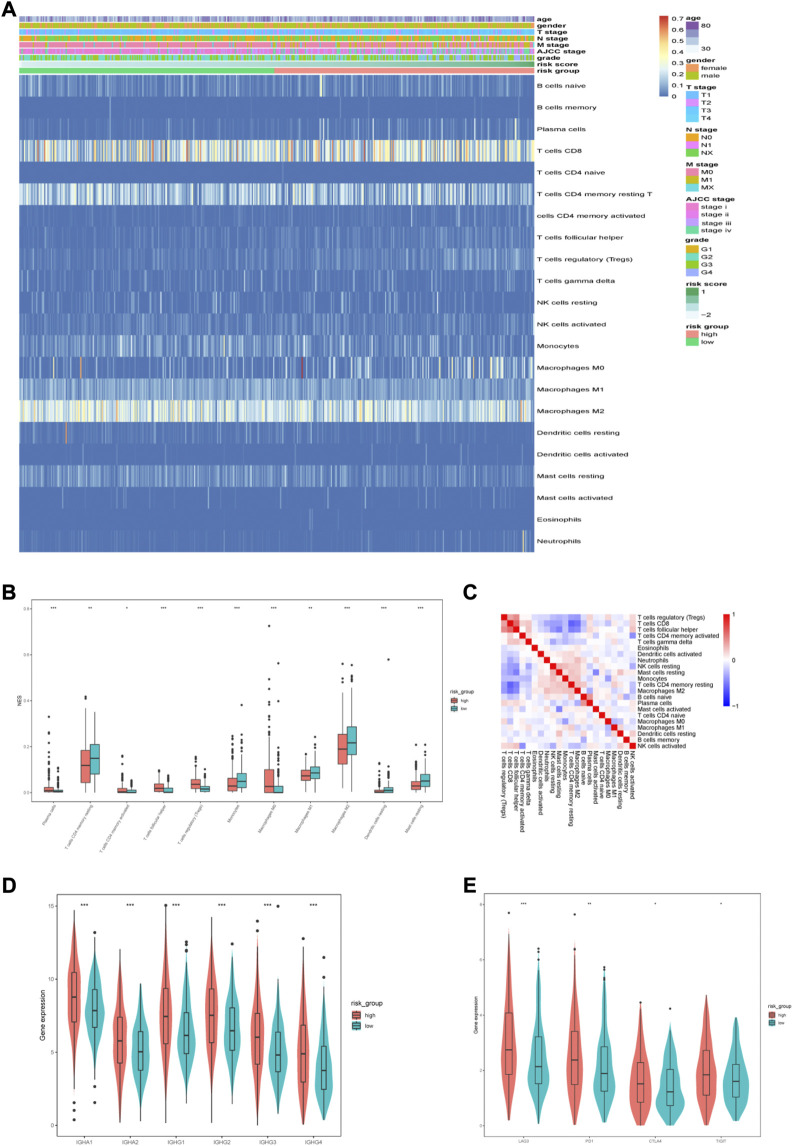
Immune characteristics of different risk groups. **(A)** Heatmap of the distribution of infiltrated immune cells and clinicopathological characteristics between the low-and high-risk groups. **(B)** Differentially infiltrated immune cells between the two risk groups. **(C)** Correlation analysis between infiltrated immune cells. **(D)** The expression of immunoglobulin in different risk groups. **(E)** The expression of ICPs in different risk groups.

A heatmap was generated to illustrate the immune infiltration landscape by different risk groups and clinicopathological features (Figure 10A). Because anti-tumor immune infiltration is critical, we analyzed the level of infiltration of immune cells. We observed significant differences in the immune cell infiltration levels between the two risk groups. In the high-risk group, there were higher infiltration levels of plasma cells, follicular helper T cells, Treg cells, and M0 macrophages (Figure 10B). The correlation analysis between immune cells is shown in Figure 10C. We also analyzed the expression levels of common immunoglobulins in different subgroups. The high-risk group had higher expression levels of IGHA1, IGHA2, IGHG1, IGHG2, IGHG3, and IGHG4 (Figure 10D). Furthermore, we analyzed the correlation between risk scores and common immune checkpoint (ICP) proteins, such as PD-1, CTLA4, TIGIT, and LAG3. Consistent with the higher Treg cell infiltration in the high-risk group, the expression of several common ICP proteins significantly increased in the high-risk group (Figure 10E). In addition, the TIDE scores were significantly higher in the high-risk group compared with the low-risk group. (Supplementary Figure 1).

### 3.7 Drug sensitivity analysis

**FIGURE 11:**
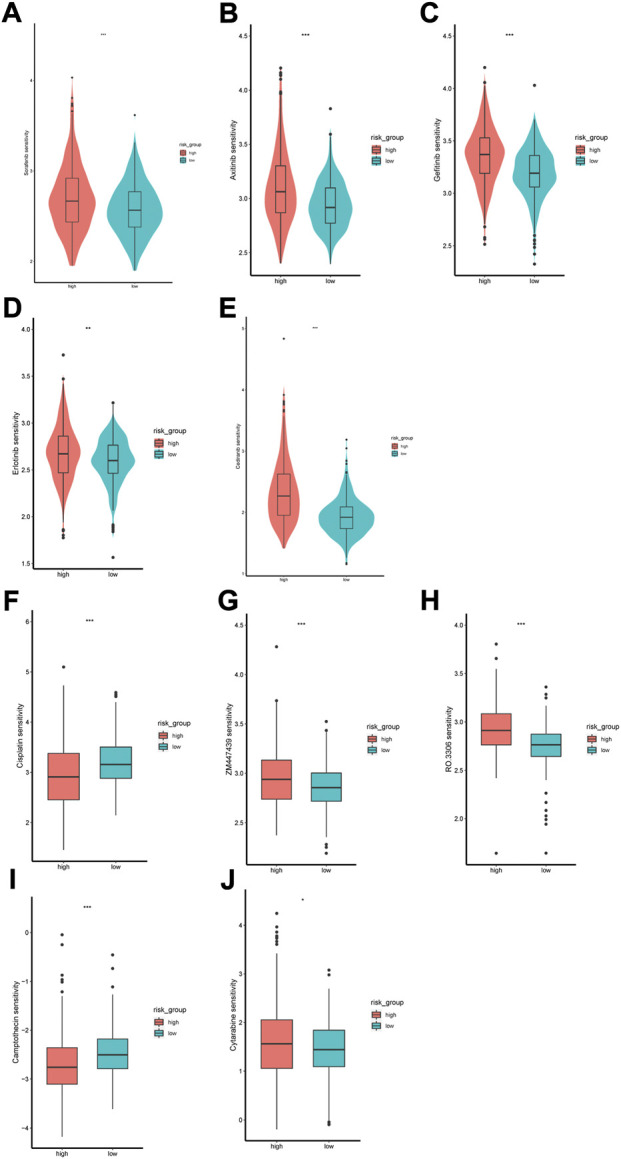
Difference of drug sensitivity. **(A–E)** Differences in response to Sorafenib, Axitinib, Gefitinib, Erlotinib and Cediranib between the two risk groups. **(F–J)** Differences in response to Cisplatin, ZM447439, RO.3306, Camptothecin and Cytarabine between the two risk groups.

The GDSC database was used to predict the sensitivity of the two groups to anti-tumor drugs. We compared the sensitivity of the two risk groups to sorafenib, axitinib, gefitinib, eroltinib, and cediranib, and we found greater sensitivity to these drugs in the low-risk group (Figures 11A–E). This might imply a favorable outcome for patients in the low-risk group with these drugs. We also evaluated several other chemotherapies that have therapeutic potential in patients with ccRCC. The high-risk group showed greater sensitivity to cisplatin and camptothecin, whereas the low-risk group showed greater sensitivity to cytarabine, ZM447439, and RO-3306 (Figures 11F–J).

### 3.8 Experimental verification

**FIGURE 12:**
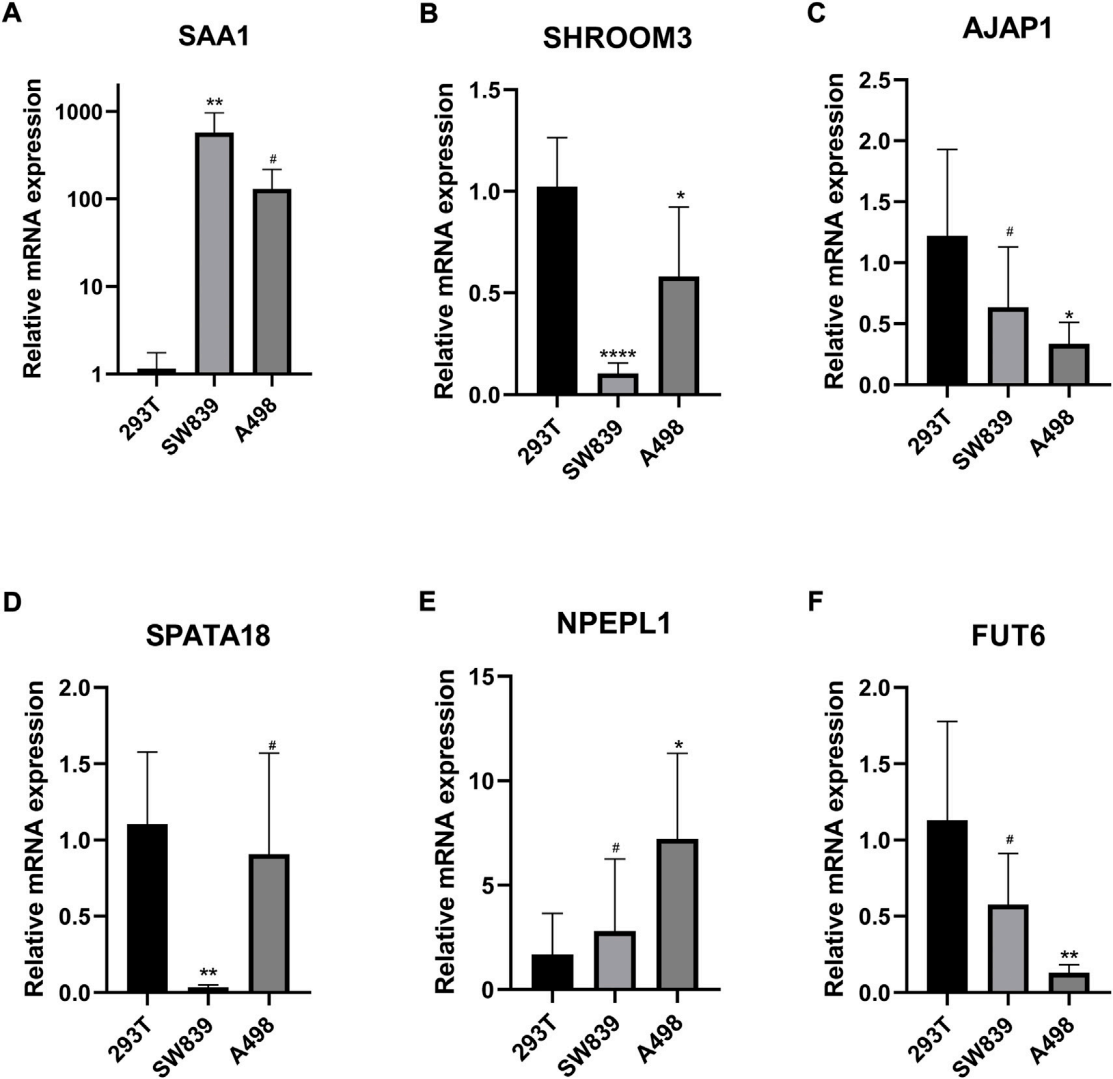
**(A–F)** Validation of the expression levels of the methylation-driven genes at the cellular level. *p < 0.05, **p < 0.01, ***p < 0.001, ****p < 0.0001, #*p* > 0.05.

We performed qRT-PCR to validate the expression of 6 MDGs in 293T cells and two ccRCC cell lines (SW839 and A498). The results showed that SAA1 and NPEPL1 were significantly upregulated in ccRCC cells, while SHROOM3, AJAP1, SPATA18, and FUT6 were significantly downregulated in ccRCC cells. (Figures 12A–F).

## 4 Discussion

ccRCC is the most common subtype of renal cell carcinoma and varies in clinicopathological features, genetic variants, DNA methylation profiles, and multi-omics features (Cancer Genome Atlas Research, 2013). Traditional TNM staging poorly reflects the individual’s tumor heterogeneity, and thus it cannot accurately predict the clinical outcome of patients. Given this, there is a great need to find effective prognostic biomarkers for ccRCC patients’ survival prediction. Epigenetics, especially DNA methylation, may influence the pathogenesis of ccRCC (Lasseigne et al., 2014; Tian et al., 2014; Evelonn et al., 2016). Therefore, we evaluated prognosis-related methylation-driven genes for their ability to guide individual therapies and improve long-term outcomes in ccRCC.

In our study, we identified MDGs by MethylMix, which has been applied to identify MDGs in various cancers (Xu et al., 2019b; Long et al., 2019). We identified and analyzed 560 MDGs in ccRCC tissues and used LASSO and multivariate Cox regression analyses to form a risk score model. We divided the patients into high- and low-risk groups according to the median risk scores, and KM curve analysis indicated that the high-risk group had a worse prognosis. We then established a nomogram that included age, M stage and the risk score for OS prediction in patients with ccRCC. The C-index, tdROC, and calibration plots showed that our predictive model performed well.

We identified six methylation-driven genes (*SAA1*, *FUT6*, *SPATA18*, *SHROOM3*, *AJAP1*, and *NPEPL1*) that are associated with ccRCC prognosis. Each of these genes have been researched in ccRCC or in other cancer types. Serum amyloid A1 (SAA1) belongs to the serum amyloid A apolipoprotein family. Inflammation, trauma, surgery, and advanced malignancy can increase SAA1 levels (Cheng et al., 2018). SAA1 knockdown in ccRCC cells inhibits tumor migration and invasion, and high *SAA1* expression predicts poor prognosis (Li et al., 2021). FUT6 is a member of the fucosyltransferase (FUT) family, which promotes tumor metastasis, proliferation, and poor prognosis (Yan et al., 2015). A recent study has shown that FUT6 promotes the proliferation, migration and invasion colorectal cancer cells (Liang et al., 2017). However, in breast cancer cells, low expression of FUT6 regulated by miR-106 b contributes to tumor migration, invasion, and proliferation (Li et al., 2016). The high-risk group had lower *FUT6* expression levels, and further studies are needed to elucidate the role of *FUT6* in ccRCC. Adherens turbine-associated protein 1 (AJAP1) is a transmembrane protein in epithelial cell adhesion junctions (Bharti et al., 2004) and has shown tumor inhibition activity in hepatocellular carcinoma (Han et al., 2017), esophageal cancer (Tanaka et al., 2015), and breast cancer (Xu et al., 2019a). Spermatogenesis-related protein 18 (SPATA18) is involved in mitochondrial quality control and induces mitochondrial-directed apoptosis in breast cancer cells (Gaowa et al., 2018). *SPATA18* expression can predict favorable clinical outcomes in colorectal cancer (Sugimura-Nagata et al., 2022). There are few reports available on aminopeptidase-like 1 (NPEPL1) and shroom family member 3 (SHROOM3); however, Shen and colleagues found that *NPEPL1* can act as an oncogene in colorectal cancer (Shen et al., 2021). Since *NPEPL1* and *SHROOM3* can reflect the prognosis of patients with ccRCC, it is important to further study their roles.

To analyze the molecular features of different risk groups, we first constructed a co-expression network *via* WGCNA, identified the module most associated with risk scores, and screened four hub genes in the modules. Low expression of four genes (*LIFR*, *AMOT*, *ITGA6*, and *EPB41L5*) suggested a poor prognosis in patients with ccRCC. Furthermore, GSEA analysis in the two risk groups indicated that multiple functions and pathways associated with immune were enriched in the high-risk group, such as complement activation, the B cell receptor signaling pathway, positive regulation of B cell activation, humoral immune response mediated by circulating immunoglobulin, and phagocytosis recognition. This finding suggests that immune cell infiltration is involved in ccRCC progression. Analysis of immune cell infiltration and the GSEA results were consistent, with plasma cells, follicular helper T cells, and Tregs being more abundant in the high-risk group. Treg cells are an important subtype of CD4^+^ helper T cells that can suppress antitumor immune responses and promote tumor progression (Binnewies et al., 2019). Given the high infiltration of plasma and follicular helper T cells, we compared the expression levels of common immunoglobulins in the two groups and found higher levels of IGHA1, IGHA2, IGHG1, IGHG2, IGHG3, and IGHG4 in the high-risk group. Serum IgA levels correlate with immune escape and tumor burden (Peppas et al., 2020), and high IgA levels indicate poor prognosis in melanoma (Bolotin et al., 2017). In addition, IgG can promote the progression of liver cancer and ccRCC (Sheng et al., 2016; Wei et al., 2019). We also explored the correlation between common ICP proteins and risk scores and found that the group with high risk had higher expression levels of ICP proteins (LAG3, PD-1, CTLA4, and TIGIT), thus suggesting a better response to immune checkpoint inhibitor treatment.

Treatment of patients with advanced ccRCC relies on immunotherapy, targeted therapy, and chemotherapy. However, tumor microenvironment alterations can lead to resistance to immune-targeted drugs in ccRCC (Tang et al., 2021); therefore, we aimed to identify drugs to which the cancer may be sensitive to help guide treatment decisions. We found drug sensitivity of the low-risk group to sorafenib, axitinib, gefitinib, erlotinib, cediranib, ZM447439, RO-3306, and cytarabine higher, and the high-risk group showed greater sensitivity to cisplatin and camptothecin. For metastatic ccRCC, Sorafenib is a first-line treatment (Escudier et al., 2007). Axitinib can be used as a second-line treatment for metastatic ccRCC or in combination with pembrolizumab or avelumab as a first-line treatment (Ingels et al., 2022). ZM447439 is a novel Aurora kinase inhibitor that induces apoptosis in rectal cancer cells through the mitochondrial apoptosis pathway (Li et al., 2010). RO-3306 disrupts the proliferation of advanced gastrointestinal stromal tumor cells by inhibiting cyclin-dependent kinase 1 (Lu et al., 2021). Our drug sensitivity analysis can provide a reference for ccRCC drug selection and may provide directions for further therapeutic target exploration.

Our study has some limitations in both approach and the information selected. First, the development and evaluation of this predictive model were based on publicly available datasets. Large-sample, multi-center, prospective clinical cohort studies are needed for further confirmation of our model. Second, the biological mechanisms by which these MDGs affect ccRCC prognosis need further investigation through *in vitro* and *in vivo* experiments.

## 5 Conclusion

We established an accurate predictive nomogram based on six methylation-driven genes for prognostic prediction in patients with ccRCC. In addition, we further analyzed the molecular characteristics, immune characteristics, and drug sensitivity of two risk subgroups. The high-risk group showed immune cell infiltrations that protect the tumor, higher tumor mutational burden, and different drug sensitivities than compared to the low-risk group. Our study provides novel insights into the epigenetic mechanisms involved in ccRCC progression and guidance for future individualized treatment of ccRCC.

## Data Availability

The original contributions presented in the study are included in the article/Supplementary Material, further inquiries can be directed to the corresponding author.

## Abbreviations

ccRCC, clear cell renal cell carcinoma; OS, overall survival; TCGA, The Cancer Genome Atlas; MDGs, methylation-driven genes; GO, Gene Ontology; tdROC, time-dependent receiver operating characteristic curve; GDSC, Genomics of Drug Sensitivity in Cancer; C-index, consistency index; WGCNA, weighted gene correlation network analysis; GSEA, gene set enrichment analysis; DEGs, differentially expressed genes; TMB, tumor mutation burden; ICP, immune checkpoint.
