# Supplementary material for: A novel prognostic model based on six methylation-driven genes predicts overall survival for patients with clear cell renal cell carcinoma
Source: Front Genet. 2022 Oct 18;13:996291. doi: 10.3389/fgene.2022.996291 (PMC9623106; doi:10.3389/fgene.2022.996291)
Supplement: Supplementary file 1 [file Table1.DOCX]

Supplementary Material

# Supplementary Figures and Tables

## Supplementary Figures

### Supplementary Figure 1.


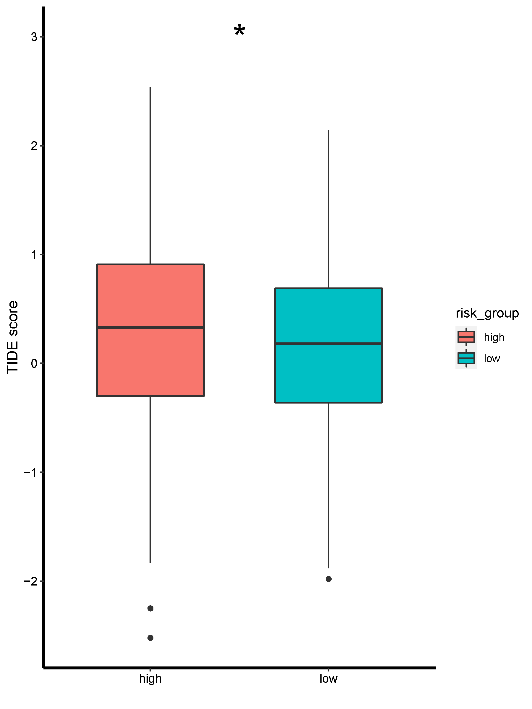


Figure S1. TIDE scores between the high-risk group and low-risk group. * P<0.05

## Supplementary Tables

### Supplementary Table 1 Primer sequences of related genes for RT-qPCR.

| Gene |  | Sequence (5’- 3’) |
| --- | --- | --- |
| SAA1 | Forward | TCGTTCCTTGGCGAGGCTTTTG |
|  | Reverse | AGGTCCCCTTTTGGCAGCATCA |
| AJAP1 | Forward | GAGACTGAGTTCATCGCCTGG |
|  | Reverse | CGTAAACGCCCGGAAATGTG |
| SHROOM3 | Forward | GGCTTTGGTCTTGTGAAGGATCC |
|  | Reverse | GAACGGAGGAATCACCAATGGC |
| SPATA18 | Forward | CATCGACAAGGCTGAGACCGTT |
|  | Reverse | CCACATAAGATGGTGTCAACGATT |
| NPEPL1 | Forward | GGCATCGTCTATGACACTGGAG |
|  | Reverse | CAGAACACAGCGTGGAGGTTGT |
| FUT6 | Forward | GCGTGTGTCTCAAGACGATCC |
|  | Reverse | GGAAGCGGGACCCATTAGG |
| GAPDH | Forward | GTCTCCTCTGACTTCAACAGCG |
|  | Reverse | ACCACCCTGTTGCTGTAGCCAA |

###

### Supplementary Table 2 Univariate and Multivariate Cox regression analyses in external validation cohort.

|  |  | Univariate Cox regression | | | | Multivariate Cox regression | | | |
| --- | --- | --- | --- | --- | --- | --- | --- | --- | --- |
|  |  |  | 95% CI | |  |  | 95% CI | |  |
|  |  | HR | Lower | Upper | *P* | HR | Lower | Upper | *P* |
| Risk score |  | 2.623 | 1.715 | 4.011 | <0.001 | 1.762 | 1.066 | 2.913 | 0.027 |
| Age |  | 1.045 | 1.003 | 1.088 | 0.0346 | 1.023 | 0.983 | 1.064 | 0.273 |
| Gender | female | reference |  |  |  |  |  |  |  |
|  | male | 2.366 | 0.703 | 7.967 | 0.164 |  |  |  |  |
| T stage |  | 2.575 | 1.674 | 3.96 | <0.001 | 1.606 | 0.954 | 2.704 | 0.075 |
| N stage | N0 | reference |  |  |  | reference |  |  |  |
|  | N1 | 65.36 | 13.473 | 317.11 | <0.001 | 13.824 | 2.374 | 80.503 | 0.003 |
|  | N2 | 13.27 | 4.188 | 42.04 | <0.001 | 4.92 | 1.376 | 17.59 | 0.014 |
| M stage | M0 | reference |  |  |  | reference |  |  |  |
|  | M1 | 6.245 | 2.626 | 14.85 | <0.001 | 2.427 | 0.815 | 7.228 | 0.111 |
